# Long-term survival from progressive multifocal leukoencephalopathy in living-donor liver transplant recipient with preformed donor-specific antibody

**DOI:** 10.1007/s13365-023-01171-x

**Published:** 2023-09-05

**Authors:** Shuhei Egashira, Akatsuki Kubota, Toshiyuki Kakumoto, Reiko Kawasaki, Risa Kotani, Kaori Sakuishi, Atsushi Iwata, Sung Kwan Bae, Nobuhisa Akamatsu, Kiyoshi Hasegawa, Mariko Tanaka, Kazuo Nakamichi, Masayuki Saijo, Tatsushi Toda

**Affiliations:** 1 Department of Neurology, Graduate School of Medicine, The University of Tokyo, 7-3-1, Hongo, Bunkyo-ku, Tokyo, 113-8655 Japan; 2 Artificial Organ and Transplantation Division, Department of Surgery, The University of Tokyo, 7-3-1, Hongo, Bunkyo-ku, Tokyo, 113-8655 Japan; 3 Hepato-Biliary and Pancreatic Surgery Division, Department of Surgery, Graduate School of Medicine, The University of Tokyo, 7-3-1, Hongo, Bunkyo-ku, Tokyo, 113-8655 Japan; 4 Department of Pathology, Graduate School of Medicine, The University of Tokyo, 7-3-1, Hongo, Bunkyo-ku, Tokyo, 113-8655 Japan; 5 Department of Virology 1, National Institute of Infectious Diseases, 1-23-1, Toyama, Shinjuku-ku, Tokyo, 162-8640 Japan

**Keywords:** Progressive multifocal leukoencephalopathy, Liver transplant, Donor-specific antibody, JC virus, Graft rejection

## Abstract

Intensive immunosuppression has enabled liver transplantation even in recipients with preformed donor-specific antibodies (DSA), an independent risk factor for graft rejection. However, these recipients may also be at high risk of progressive multifocal encephalopathy (PML) due to the comorbid immunosuppressed status. A 58-year-old woman presented with self-limited focal-to-bilateral tonic-clonic seizures 9 months after liver transplantation. She was desensitized using rituximab and plasma exchange before transplantation and was subsequently treated with steroids, tacrolimus, and everolimus after transplantation for her preformed DSA. Neurological examination revealed mild acalculia and agraphia. Cranial MRI showed asymmetric, cortex-sparing white matter lesions that increased over a week in the left frontal, left parietal, and right parieto-occipital lobes. Polymerase chain reaction (PCR) of the cerebrospinal fluid for the JC supported the diagnosis of PML. Immune reconstitution by reducing the immunosuppressant dose stopped lesion expansion, and PCR of the cerebrospinal fluid for the JC virus became negative. Graft rejection occurred 2 months after immune reconstitution, requiring readjustment of immunosuppressants. Forty-eight months after PML onset, the patient lived at home without disabling deficits. Intensive immunosuppression may predispose recipients to PML after liver transplantation with preformed DSA. Early immune reconstitution and careful monitoring of graft rejection may help improve outcomes.

## Introduction

Progressive multifocal leukoencephalopathy (PML) is an opportunistic infection of the central nervous system caused by the John Cunningham virus (JCV) (Berger et al. 2013). Although initially described in patients with human immunodeficiency virus (HIV), PML can also occur after solid organ transplantation (Molloy and Calabrese 2009). PML is rare following liver transplantation, with a reported incidence of 0.21–0.76% (Martinez and Ahdab-Barmada 1993; Bronster et al. 1995). The prognosis of non-HIV PML is poor, with an estimated life expectancy of three months (Bloomgren et al. 2012).

Donor-specific antibodies (DSA) are antibodies against the donor-derived human leukocyte antigen (HLA) (Demetris et al. 2016). Because DSA are an independent risk factor for graft rejection, liver transplantation with preformed DSA has been discontinued (Demetris et al. 2016). Intensive immunosuppression has recently enabled liver transplantation in recipients with preformed DSA; however, PML risk remains high for recipients due to the comorbid immunosuppressed status (Akamatsu et al. 2021). We describe a case of long-term survival from PML after liver transplantation for preformed DSA. This report provides insights into this potentially manageable complication of liver transplantation with preformed DSA.

## Case report

A 58-year-old, right-handed woman underwent living-donor liver transplantation for decompensated alcoholic liver cirrhosis. She had performed DSA, was desensitized with rituximab, and had undergone plasma exchange before transplantation. The patient received methylprednisolone (4 mg/day), tacrolimus (3 mg/day), and everolimus (1.5 mg/day).

Nine months after liver transplantation, she was admitted to our hospital with self-limited focal-to-bilateral tonic-clonic seizures. On admission, a neurological examination revealed mild acalculia and agraphia. She had no history of headache, fever, infection, or vaccination. CD4-positive and CD8-positive T cell counts were 185/μL and 1383/μL, respectively.

**Fig. 1:**
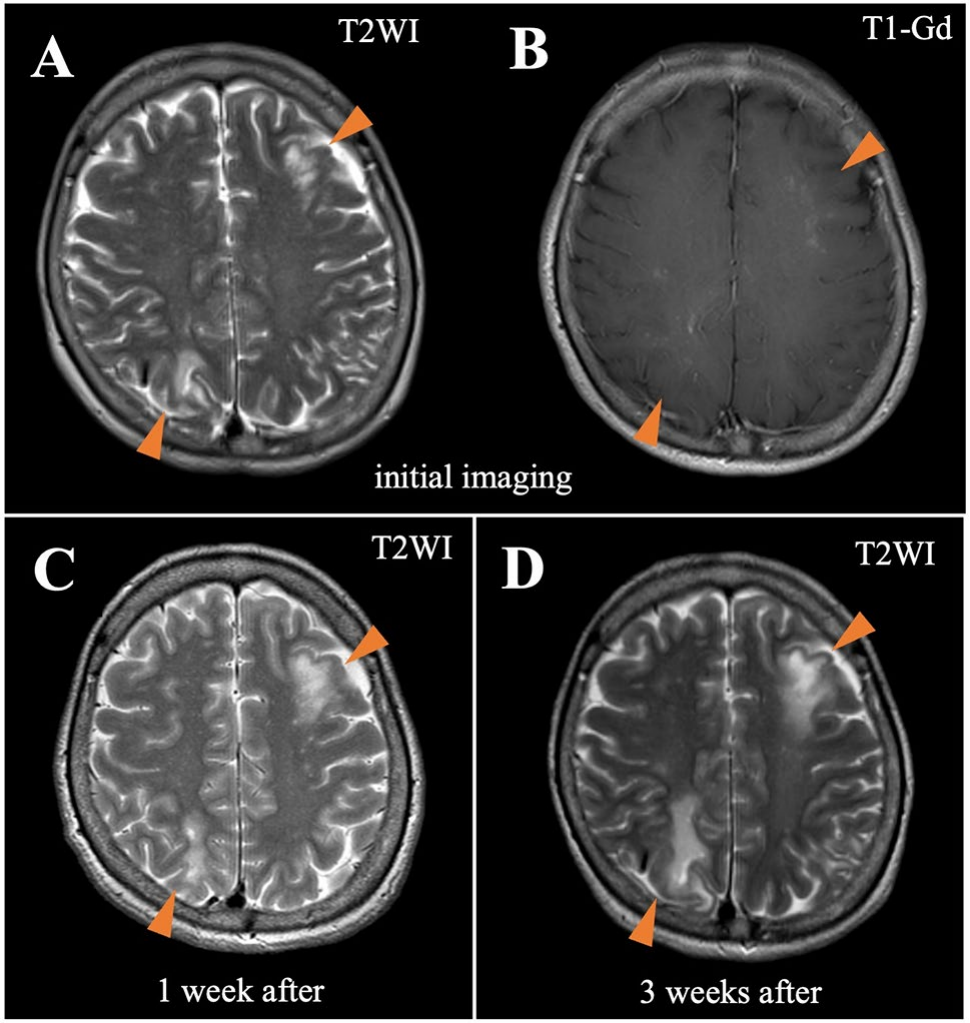
Magnetic resonance imaging of the head. T2WI indicates T2-weigted image; T1-Gd, gadolinium-enhanced T1-weighted image. T2-weighted image at the onset of progressive multifocal encephalopathy (PML) shows asymmetric, cortex-sparing white matter lesions in the left frontal and right parieto-occipital lobes (**A**). Gadolinium-enhanced T1-weighted image shows partial enhancement in these brain lesions (**B**). The lesions enlarge weekly, with a confluent pattern (**C**, 1 week after initial imaging; **D**, 3 weeks after initial imaging)

T2-weighted cranial magnetic resonance imaging (MRI) showed asymmetric, cortex-sparing white matter lesions in the left frontal, left parietal, and right parieto-occipital lobes, with weekly enlargements. Gadolinium-enhanced T1-weighted image showed partial enhancement in these brain lesions (Fig. 1A–C). Electroencephalography revealed repetitive spikes localized in the right parietal region (P4 max). Cerebrospinal fluid (CSF) examination showed no cells, a protein concentration of 33 mg/dL, an IgG index of 0.40, and positive oligoclonal IgG bands. JCV DNA was detected in the CSF using quantitative polymerase chain reaction (PCR) (166 copies/mL) at the National Institute of Infectious Diseases (Tokyo, Japan). Epstein–Barr virus, herpes simplex virus, cytomegalovirus, and varicella zoster virus were not detected by PCR. Serum and cerebrospinal fluid tests for *Mycobacterium tuberculosis*, Cryptococcus, syphilis, and HIV were also negative.

The patient fulfilled the diagnostic criteria for definite PML (Berger et al. 2013). Immune reconstitution was initiated before confirming the PCR findings. Tacrolimus was discontinued, and the methylprednisolone and everolimus doses were gradually tapered. There was no neurological deterioration, and the JCV DNA titer was reduced from 166 to 18 copies/mL 1 week after initiating immune reconstitution. As the lesions enlarged, the treatment was switched from prednisolone and everolimus to cyclosporine alone (50 mg/day) (Fig. 1D). Mirtazapine (15 mg/day) and mefloquine (275 mg/week) were administered after approval from the institutional ethics committee. Three weeks after initiating immune reconstitution, lesion expansion ceased. Monthly tests for JCV DNA in the CSF were also negative. There was no evidence of immune reconstitution inflammatory syndrome.

Two months after initiating immune reconstitution, aspartate transaminase (AST) and alanine transaminase (ALT) levels increased to 40–60 IU/L. Liver biopsy revealed portal inflammation with lymphocytes and eosinophils, bile duct damage, and endothelialitis with bile ductular proliferation. C4d immunohistochemical staining was positive in the portal veins. Acute cellular and antibody-mediated rejection was suspected. Her immunosuppressant dose was gradually increased to 2 mg/day methylprednisolone, 1.5 mg/day everolimus, and 75 mg/day cyclosporine, with close monitoring of neurological symptoms and MRI. Subsequently, her AST and ALT levels normalized with no PML relapse.

Forty-eight months after PML onset, she lived at home with an Expanded Disability Status Scale score of 1.5. MRI performed every 6 months showed no lesion enlargement. She remained negative on PCR for CSF JCV for over 3 years and 7 months (16 PCR tests). Graft dysfunction was not observed.

## Discussion

We describe a case of long-term survival following PML in a liver transplant recipient with preformed DSA. To the best of our knowledge, this is the first reported case of preformed DSA among post-liver transplant patients with PML in the PubMed database (Table 1).

**Table 1:** Reported cases of progressive multifocal leukoencephalopathy after liver transplantation

**Year of publication**	**Author**	**Age, year**	**Sex**	**DSA**	**IS**	**Time from transplant to PML onset, month**	**Treatment for PML**	**Graft rejection during PML treatment**	**Prognosis**	**Time from PML diagnosis to death or survival, month**
1994	Worthmann F	53	Female	-	steroids, CsA, AZA, TAC	1.5	None	NA	Death	NA
1995	Aksamit AJ Jr	55	Female	-	steroids, CsA	NA	None	NA	Death	NA
1995	Bronster D	51	Male	-	steroids, CsA, AZA	1.5	Tapering IS, AraC	-	Death	0.5
2001	Boulton-Jones JR	60	Male	-	steroids, CsA, AZA, TAC, MMF	11	Tapering IS, AraC	-	Death	60
2005	Lima MA	39	Female	-	steroids, CsA, TAC, MMF, BXM	8	Tapering IS, AraC	-	Death	1.5
2005	Alibert S	59	Female	-	Steroids, TAC	18	Tapering IS, AraC, PegIFN	-	Death	5
2009	Yehia B	66	Female	-	MMF	113	Tapering IS	-	Death	NA
2011	Verhelst X	72	Female	-	TAC, MMF, BXM	34	Tapering IS	-	Death	4
2011	Mateen F	42	Female	-	NA	1	NA	NA	Survival	155
2011	Mateen F	56	Female	-	NA	1	NA	NA	Death	9
2011	Mateen F	68	Male	-	NA	8	NA	NA	Death	9
2011	Mateen F	56	Female	-	NA	116	NA	NA	Death	124
2015	Ozdemir F	55	Male	-	Steroids, TAC, MMF	9	Tapering IS, AraC	-	Death	2
2015	Yoshida T	66	Male	-	TAC, SLR	48	Mefloquine	+	Death	23
2016	Dumortier J	48	Female	-	Steroids, TAC, MMF	144	Tapering IS	+	Death	26
2016	Dumortier J	54	Male	-	Steroids, TAC	204	Tapering IS	-	Survival	36
2017	Moreno-Estébanez A	76	Female	-	Steroids, TAC, MMF	132	Tapering IS, mirtazapine	-	Death	2.25
2017	Avsenik J	65	Male	-	Steroids, TAC, MMF	4	Tapering IS, mirtazapine, cidofovir	-	Survival	4.75
2019	Ahmadinejad Z	41	Male	-	TAC, MMF	2	Tapering IS	-	Survival	7
2019	Rastogi A	59	Male	-	Steroids, TAC, MMF	2	Tapering IS	-	Death	0.75
2023	Present case	59	Female	+	Steroids, TAC, MMF, RTX, PE, EVL	9	Tapering IS, mirtazapine, mefloquine	+	Survival	48

*DSA* donor-specific antibody, *IS* immunosuppressant, *CyA* cyclosporine A, *AZA* azathioprine, *TAC* tacrolimus, *MMF* mycophenolate mofetil, *BXM* basiliximab, *SLR* sirolimus, *RTX* rituximab, *PE* plasma exchange, *EVL* everolimus, *PML* progressive multifocal encephalopathy, *AraC* cytosine arabinoside, *PegIFN* pegylated interferon, *NA* not applicable

In Japan, liver transplantations from brain-dead donors remain low. The alternative option is living-donor liver transplantation, often with organs donated by their relatives. However, due to HLA similarity, the recipient is likely to have DSA and be at high risk of rejection (Yoshizawa et al. 2013). Intensive immunosuppression mitigates the risk of rejection, but it may also increase the risk of PML due to the comorbid immunosuppressed status (Yoshizawa et al. 2013). Among immunosuppressants, rituximab remains high-risk factor for PML (Clifford et al. 2011). Patients with liver cirrhosis without overt immunosuppression may develop PML (Gheuens et al. 2010). Desensitization in renal transplants is not associated with JCV viremia (Toyoda et al. 2015). However, they did not evaluate CSF, and the results may not be extrapolated to liver transplantation. PML should be considered a neurological complication in liver transplant recipients with DSA.

Long-term survival of patients with PML after liver transplantation has rarely been reported. Eighty percent of patients with post-liver transplant PML died within a median time of 5 months from diagnosis to death (interquartile range, 2–23 months) (Table 1). In non-HIV-PML, immune reconstitution in the early phase is linked to favorable outcomes (Amend et al. 2010). However, tapering immunosuppressant poses a dilemma for graft rejection in patients with PML after organ transplantation. Since the initial immunosuppressive dose reduction alone was not sufficient to stop the lesion expansion completely, we tried mirtazapine and mefloquine. It should be noted, however, that there is no evidence for the efficacy of these drugs, and there is an opposing viewpoint that they may only expose the patient to unnecessary side effects (Clifford et al. 2013). In post-liver transplant PML, rejection may be more likely to occur in DSA-positive cases than in DSA-negative cases, among which rejection occurred in 20% of the patients during PML treatment (Table 1).

In conclusion, intensive immunosuppression by preformed DSA may predispose recipients to PML after liver transplantation. Early immune reconstitution and careful monitoring of graft rejection may improve outcomes.

## Data Availability

The datasets analyzed during the current study are available from the corresponding author on reasonable request.
